# Climate change, range shifts, and the disruption of a pollinator-plant complex

**DOI:** 10.1038/s41598-019-50059-6

**Published:** 2019-10-01

**Authors:** Emma P. Gómez-Ruiz, Thomas E. Lacher Jr.

**Affiliations:** 1 Universidad Autónoma de Nuevo León, Facultad de Ciencias Biológicas, Ave. Universidad S/N, San Nicolás de los Garza, Nuevo León, MX 66455 Mexico; 2 Department of Wildlife and Fisheries Sciences, Texas A&M University, 534 John Kimbrough Blvd., TAMU 2258, College Station, TX 77843-2258 USA

**Keywords:** Conservation biology, Ecological modelling, Climate-change ecology, Ecological modelling

## Abstract

Climate change has significant impacts on the distribution of species and alters ecological processes that result from species interactions. There is concern that such distribution shifts will affect animal-plant pollination networks. We modelled the potential future (2050 and 2070) distribution of an endangered migratory bat species (*Leptonycteris nivalis*) and the plants they pollinate (*Agave* spp) during their annual migration from central Mexico to the southern United States. Our models show that the overlap between the *Agave* and the endangered pollinating bat will be reduced by at least 75%. The reduction of suitable areas for *Agave* species will restrict the foraging resources available for the endangered bat, threatening the survival of its populations and the maintenance of their pollination service. The potential extinction of the bat *L. nivalis* will likely have negative effects on the sexual reproduction and genetic variability of *Agave* plants increasing their vulnerability to future environmental changes.

## Introduction

Climate is one of the main factors determining the geographic distribution of species^1^ and combined with patterns of land-use change can result in the contraction or shift in species ranges^2,3^. Studies have shown that, under future climate scenarios, the suitable areas for many species would change, reducing or increasing in size or shifting in latitude and elevation^4–7^. There is concern that these changes will increase extinction risk^8^ and affect biotic interactions^9^. Additional evidence shows that changes in climate are affecting both insect phenology^10^ and plant phenology, in the latter case delaying flowering periods and causing a mismatch with the presence of key migratory pollinators^11–13^. The loss of a single pollinator can disrupt an entire pollination network by affecting patterns of specialization and floral fidelity of other pollinators^14,15^. Thus, shifts in temperature and precipitation regimes under current climate change scenarios might place plant-pollinator mutualisms under a complex mix of spatial and temporal threats.

Concerns about the loss of pollinators has increased calls for the inclusion of the risk factors of climate change into vulnerability assessments of extinction risk across multiple taxa^16^ and in particular for the integration of climate change assessments into the IUCN Red List process^17^. For species already listed as endangered (EN) under pre-climate change assessments, the incorporation of climate data might well indicate increased extinction risk in many cases.

Plants of the genus *Agave*, subgenus *Agave*, (hereafter referred to simply as “agaves”) have evolved flower traits (paniculate flowering) that reflect convergent adaptation for pollination by bats^18^, in particular their key pollinators, *Leptonycteris spp*. Compared with other pollinators, bats are large-bodied and can carry greater pollen loads across distant populations of agaves^19^. Previous studies show evidence that long-nosed bats (*Leptonycteris* spp.) are the most important pollinators for agaves and have influenced their rapid speciation^20–22^. Likewise, authors have suggested that the *Leptonycteris*-*Agave* interspecific relationship may be an example of coevolution and mutualism^23–25^.

The Mexican long-nosed bat (*Leptonycteris nivalis*), migrates up to 1200 km north from central Mexico to the south-western United States every spring, following the blooms of paniculate agaves^26,27^. This bat species is listed as endangered by the United States^28^, Mexico^29^, and the International Union for the Conservation of Nature (IUCN Red List)^30^ due to declines in populations of over 50% in the past ten years. The bats travel a corridor that follows the Sierra Madre Oriental in Mexico, beginning in the south-western United States, tracking areas of high agave richness^31^. Authors have pointed out that the migration of long-nosed bats is an “endangered phenomenon” because of its complexity (scarcity of roosting sites with ideal conditions and scarcity of flowering agave)^32^. Losing the *Leptonycteris-Agave* interaction has ecological and economic implications because agaves play a critical role in maintaining soil stability in arid and semi-arid ecosystems and have socio-economic value, providing food and cultural services for humans in the form of natural fibres and traditional beverages, such as tequila and mescal^23,33^.

The relationship between *L. nivalis* and the primary *Agave* species it pollinates has never been examined from a climate change perspective. The incorporation of data on potential range shifts of this pollinator and the key food resources could well indicate a more dire scenario for the persistence of *L. nivalis* over time as well as for the agave species it pollinates. We analysed potential effects of climate change on the geographic distribution of agaves and *L. nivalis* for the years 2050 and 2070, using ensemble climate models and multiple radiative forcing scenarios from the Intergovernmental Panel on Climate Change. Changes in distributions as a result of climate change could reduce the available foraging resources and affect the population viability of the endangered bat as well as the pollination service they provide.

## Results

Our models show that the suitable environments for all the species are reduced under future scenarios of climate change (Supplementary Information Fig. S1). *Agave gentryi*, *A. horrida* and *A. salmiana* are reduced more than 80% under all scenarios, as well as *A. parryi* and *A. palmeri* under three scenarios (Table 1).

**Table 1:** Percentage of no change, loss and gain in each species’ environmentally suitable area.

Species	Scenario	No Change	Loss	Gain
*Agave americana*	RCP 4.5 2050	44	56	0
	RCP 8.5 2050	39	61	0
	RCP 4.5 2070	54	43	3
	RCP 8.5 2070	23	77	0
*Agave asperrima*	RCP 4.5 2050	26	73	1
	RCP 8.5 2050	35	63	1
	RCP 4.5 2070	20	79	2
	RCP 8.5 2070	25	73	2
*Agave gentryi*	RCP 4.5 2050	14	85	0
	RCP 8.5 2050	17	82	0
	RCP 4.5 2070	11	89	0
	RCP 8.5 2070	9	91	0
*Agave havardiana*	RCP 4.5 2050	27	73	0
	RCP 8.5 2050	45	55	0
	RCP 4.5 2070	31	69	0
	RCP 8.5 2070	21	79	0
*Agave horrida*	RCP 4.5 2050	20	80	0
	RCP 8.5 2050	1	99	0
	RCP 4.5 2070	14	86	0
	RCP 8.5 2070	2	98	0
*Agave inaequidens*	RCP 4.5 2050	51	48	1
	RCP 8.5 2050	51	48	1
	RCP 4.5 2070	48	52	1
	RCP 8.5 2070	24	76	0
*Agave palmeri*	RCP 4.5 2050	26	70	5
	RCP 8.5 2050	17	80	3
	RCP 4.5 2070	6	90	4
	RCP 8.5 2070	4	93	3
*Agave parryi*	RCP 4.5 2050	9	91	0
	RCP 8.5 2050	29	69	2
	RCP 4.5 2070	9	91	0
	RCP 8.5 2070	5	95	0
*Agave salmiana*	RCP 4.5 2050	12	88	0
	RCP 8.5 2050	10	90	0
	RCP 4.5 2070	14	86	0
	RCP 8.5 2070	5	95	0
*Leptonycteris nivalis*	RCP 4.5 2050	19	79	2
	RCP 8.5 2050	34	64	1
	RCP 4.5 2070	19	80	1
	RCP 8.5 2070	22	76	2

**Figure 1:**
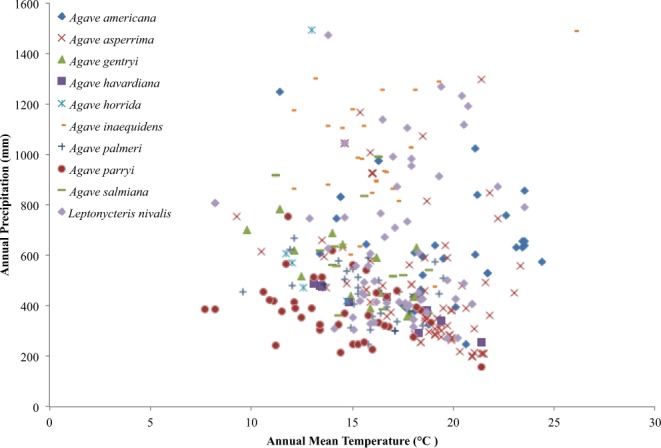
Bi-dimensional ecological distribution (annual precipitation and mean temperature) of the known occurrences of the species.

For *A. parryi*, *A. havardiana*, and *A. gentryi*, the Global Circulation Model (GCM) ensembles for 2050 show greater losses of suitable area for the low-end Representative Concentration Pathway (RCP) 4.5 than for the high-end RCP 8.5. The same trend is seen for *A. asperrima* and for the bat *L. nivalis*, but in both time projections of 2050 and 2070. Overall the tendency in the RCP 8.5 scenario is towards aridity and the occurrences of the five species mentioned in this paragraph tend to have the lowest annual precipitation values under the current conditions. The models thus indicate that less area will be loss under RCP 8.5, which is the extreme high-end scenario (Fig. 1).

**Figure 2:**
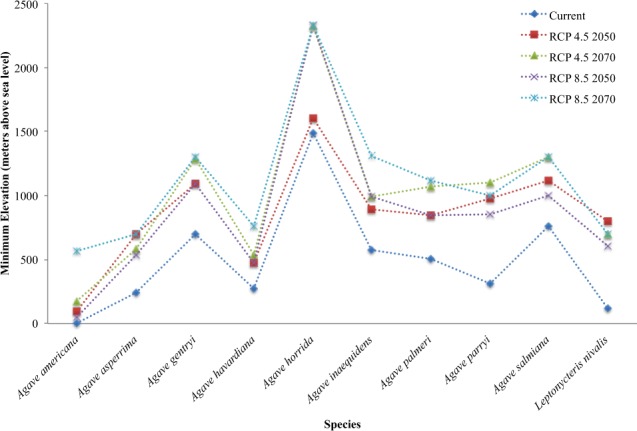
Minimum elevation in the potential distribution areas of each species.

The future models show a small expansion of suitable areas for six of the species in at least one of the RCPs and time frames. *A. palmeri* and *L. nivalis* show slight increases in suitable areas in all the scenarios. The highest gain for *A. palmeri* is 5% under RCP 4.5 and 2050 projection, and for *L. nivalis* is 2% under two different scenarios. However, the gains are trivial relative to the magnitude of the losses (Table 1). The overall trend for all the species is that the suitable areas would retreat to higher elevation areas in every future scenario considered in this study (Fig. 2).

**Figure 3:**
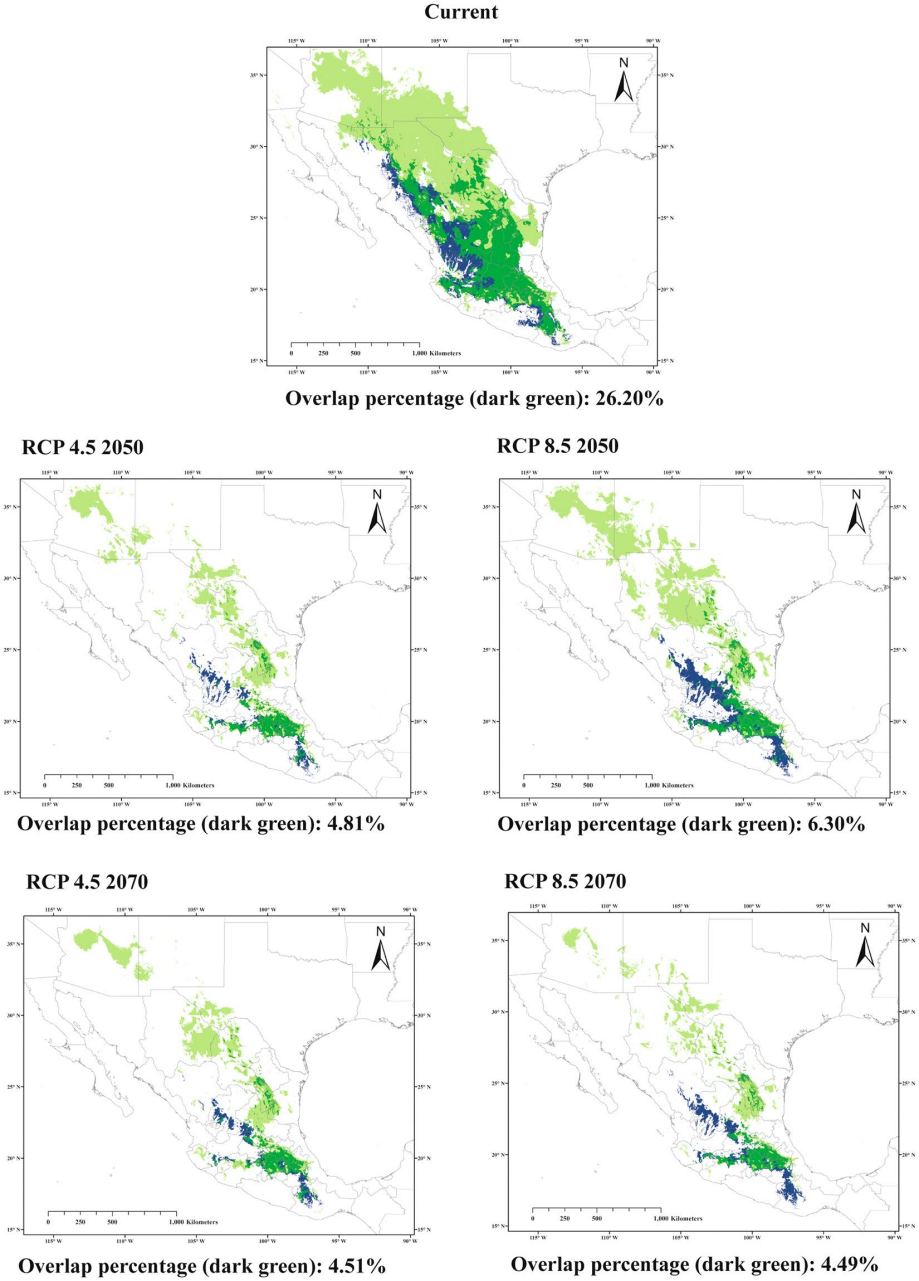
Overlap (dark green) between environmentally suitable areas for *Agave* species (light green) and *Leptonycteris nivalis* (blue).

**Figure 4:**
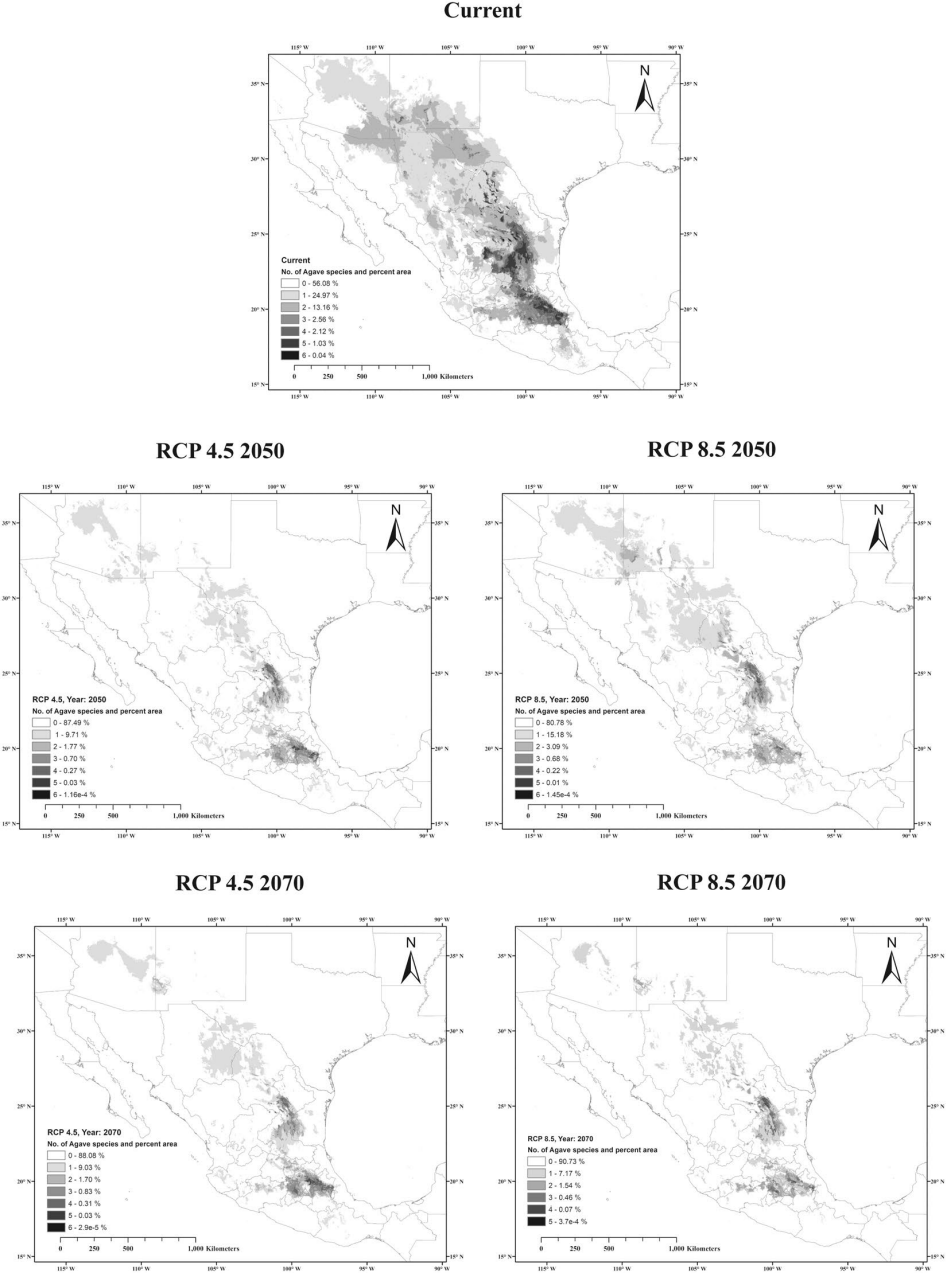
*Agave* richness patterns under current and future climate scenarios. Darker colour indicates higher number of *Agave* species.

We combined the presence area maps for all agave species into one map representing the presence of one or more agaves. The overlap of this map with the suitable area map for *L. nivalis* under current climatic conditions is 26.2% and under all the future scenarios there is at least a 75% decrease in overlap percentage relative to current distributions (Fig. 3). We also project a change in the pattern of richness with a smaller proportion of areas with one or more agave species present in future scenarios than under current climate conditions (Fig. 4).

## Discussion

We developed models based only on bioclimatic variables, as a first approximation to potential impacts of climate change on the distribution of species. Several reviews on modelling future species distributions recommend that, in addition to climate, other variables should be considered. These variables include dispersal data, genetic adaptation, species behavioural plasticity, and biotic interactions^9,34,35^. Generating this information requires years of study and there is an urgent need to guide management actions for minimizing threats to biodiversity, even more so in endangered species complexes like the *L. nivalis-Agave* interaction. Our models are not predictions of future distributions but rather indicate the direction of change in the distribution of suitable environments for the *Agave* species and their pollinating bat *L. nivalis*. In general, for all *Agave* species, most of the seeds produced fall from the fruit capsules near the parent plant, but others in strong wind may be blown several meters^23^. This suggests that agaves have a limited dispersal potential and incorporating this variable in the models will likely restrict even more the size of areas with suitable environments in future climate scenarios, increasing the risk of disaggregation of the pollination complex^15^.

Under most climate change scenarios, it is expected that suitable environments for species will be reduced to a higher degree under the extreme high-end RCP (Representative Concentration Pathway) 8.5. There were several exceptions that appear to be related to species that currently occur in the arid end of the spectrum for current distributions (Fig. 1). *A. parryi*, *A. havardiana*, and *A. gentryi* show greater losses of suitable habitat under RCP 4.5 in 2050 compared to RCP 8.5, suggesting more tolerance to more extreme climate change in the intermediate time frame. In addition, *A. asperrima* as well as *L. nivalis* exhibited less contraction of suitable area under RCP 8.5 both in 2050 and 2070. This suggests that species currently adapted to high aridity are slightly less impacted than less arid adapted species^36–38^.

Previous studies exploring the effects of future climates indicate a general trend for species retreating their ranges to higher elevation areas^5,39,40^. Our results show the same trend; all species distribution models change in a similar fashion by shifting up in elevation. A study of the thermal niche phylogenetic patterns in nectar feeding bats found that the two most cold-adapted species are *L. nivalis* and *Choeronycteris mexicana*^41^. Both occur in mountainous regions of Mesoamerica and are particularly sensitive to warming climates. *L. nivalis* in addition is derived from the Antillean clade in the subfamily, where most species have higher thermal tolerances, and thus represents a more recent adaptation for the cooler mountain regions of Mexico where it occurs.

Changes in temperatures and precipitation will also affect the species phenology in ways we do not clearly understand. There is little information about the specific cues that trigger flowering in agaves, but there is consensus that precipitation is an important variable^23,42^. We recommend implementing an annual monitoring program in the study area to document flowering timing in the agave populations and associated precipitation data. This information will help to elucidate long-term trends in the *Agave-L. nivalis* interaction that will result from changing patterns in precipitation and inform future modelling exercises.

Pollinators and plants form networks, where modifications in links can have unexpected impacts. Romo *et al*.^43^ examined the effects of climate change on several pairs of tightly linked interactions between butterflies and forage plants in Spain. Favourability indices derived from climate variables vary among the species pairs. In some cases, favourability increases or declines similarly for species pairs and in other cases butterfly and plant favourability shift in opposite directions. Local declines in interacting pairs can thus result from similar losses for both species, but also for declines in favourability for only one of the two mutually dependent species. Our situation differs in that *L. nivalis* can use several different species of agave. The presence of different species of agave along the bat migratory corridor allows for the availability of foraging resources for longer periods of time because each agave species flowers at a particular time frame and agave richness is an important component of the complex^31^. Thus, the bat can compensate on poor productivity or delayed phenology of one species by switching to another. This assumes that the removal or changes in abundance of one or more agave species does not affect the pollination preferences of the bat. Research on a network of bee pollinators and their forage flowers demonstrated that the loss of one pollinator causes shifts in the fidelity of the remaining bees, causing unexpected declines in fitness of some of the plants^14,15^. The situation with *L. nivalis* and the agaves is obviously different, however specific preferences of *L. nivalis* are not known, nor whether the loss of some of its forage plants would impact the bat’s preferences for remaining species.

Our models indicate that for some *Agave* species the overlapping area with *L. nivalis* completely disappears under future climate scenarios. Suitable areas for *A. parryi* and *A. palmeri* tend to retreat North, opposite to what the models show for *L. nivalis*, which tends to retreat South. Studies on the pollination biology of *A. palmeri* suggest bats are important pollinators for this agave species^44,45^. Our models also suggest that the pattern of agave richness dramatically changes in future scenarios, and the areas with two or more *Agave* species overlapping are greatly reduced (Fig. 4). The fewer agave species present in one region reduces the period with available flowers. This might result in foraging stress for the endangered *L. nivalis* and force them to migrate to other areas earlier. *L. nivalis* is also dependent upon caves located along the mountain corridor where it migrates^31,32^ so its ability to move will be dependent upon the availability of suitable caves for roosting.

In the long-term, the consequences of the mismatch in range shifts between *L. nivalis* and agaves might result in the reduction or disappearance of bat dependent agave populations throughout areas where they currently are present. Wild agaves are important for maintaining soil stability and preventing erosion, and their absence would affect negatively the arid and semi-arid ecosystems where they occur. More research is needed on the reproductive ecology of agave species to better understand the role of *L. nivalis* in their pollination, and document which agaves are more dependent on the presence of the bat for their successful pollination and seed production. The loss of key pollinators results in cascading effects at the ecosystem level^15^.

Two other nectar-feeding bats (*L. yerbabuenae*, and *C. mexicana*) occur within the range of *A. palmeri* and *A. parryi*. Nonetheless, *L. nivalis* would move pollen further distances because its migratory range is the largest of the three nectarivorous bat species, and the reported maximum elevation for this bat is 3 780 m which is much higher than the maximum reported for *L. yerbabuenae* and *C. mexicana* (1 800 m and 2400 m, respectively) meaning that it can pollinate agave species adapted to high elevations. Moving the pollen over long distances increases the opportunities for maintaining higher genetic diversity in the agaves, which expands the resilience potential of these plants to environmental change. Aguilée *et al*.^46^ suggest that plants receiving pollen dispersed from long distances may survive climate change because of ecological niche shifts, perhaps due to the movement of alleles from individual plants with different local climatic conditions.

The local extirpation or extinction of the bat *L. nivalis* could have a negative effect on the sexual reproduction and genetic variability of agaves, increasing their vulnerability to future environmental and climatic changes. Whether changes in the current distributions and elevational tolerances of the other two species. *L. yerbabuenae* and *C. mexicana*, might compensate for the loss of *L. nivalis* is hard to assess. Their current distributions and elevational limits differ from *L. nivalis*, as likely do their preferences for different agave species. The persistence of the *Agave*-*L. nivalis* interaction over the long-term is one component for mitigating the detrimental effects of future climate change in arid and semi-arid ecosystems of Mexico and the United States by maintaining agave species important for ecosystem function and community well-being. Maintaining this pollination corridor is critical, and adaptation measures should include careful monitoring of agave declines so that richness of native agave species is maintained in suitable areas for *L. nivalis*. Assisted migration of tolerant agave species may become a necessary response to protect this important pollination complex.

## Methods

### Study area

Our study considered the entire range of the Mexican long-nosed bat (*L. nivalis*) from central Mexico to the south-western United States (Top left: 43.520°, −116.754°; Down right: 15.645°, −95.079°). This bat migrates every spring following the blooming events of paniculated agaves^26^. Thus, we selected paniculate agaves occurring within the bat’s range and documented either in the bat’s diet^47^ or to be blooming when the bat was present in a particular area^27,48–50^.

### Species studied

There is prior research showing a correspondence between the occurrence of this bat and areas with higher number of agave species^31^. We modelled the distribution of 9 agave species occurring in the bat’s range: *A. americana, A. asperrima, A. gentryi, A. havardiana, A. parryi, A. salmiana, A. horrida, A. inaequidens, A. palmeri*.

We obtained occurrence records from three online biodiversity databases to model the distributions of the nine agave species and the bat: the Global Biodiversity Information Facility (GBIF, www.gbif.org), the Comisión Nacional para el Conocimiento y Uso de la Biodiversidad (CONABIO), and the Mammal Networked Information System (MaNIS). We also compiled records from museum and herbarium collections (CIIDIR-IPN, FCB-UANL, UAAAN), previous published research and surveys^23,24,51–56^, and our own agave and bat survey work. We only used records of agaves occurring in the wild. We consulted with experts in agave taxonomy and ecology (Socorro Gonzalez-Elizondo and Martha Gonzalez-Elizondo from Instituto Politécnico Nacional) who verified all occurrence data from the above sources and all data points were cleaned for errors. We verified the bat occurrence data and only retained records that were confirmed to be the species *L. nivalis* according to the only taxonomic review of the genus *Leptonycteris*^24^.

To address concerns over spurious results and weakened validation statistics caused by spatial autocorrelation in occurrence data, we only considered occurrences situated at least 10 km apart^57–59^. Our analysis included species with at least 5 records. For species with 20 or more records we created a random subset of points to be used later for testing model performance as recommended in species distribution modelling literature^59^. For species with less than 20 records we did not split the data for testing the model since there would not be enough records to calibrate the model; instead we used the jackknife approach presented by Pearson *et al*.^57^. This approach consists of removing each observed locality once from the set of data and building a model using the remaining n-1 localities. Predictive performance is assessed based on the ability of each model to predict the locality excluded.

### Climate data

For generating the potential future distributions of environmentally suitable areas for the species of interest, we first characterized the current (representative of 1950–2000) climatic niches using 19 bioclimatic variables obtained from WorldClim project^60^ with a resolution of 0.0083°/px (ca. 1 km^2^). These variables are derived from temperature and precipitation data and represent annual trends, seasonality and extreme conditions.

Several authors have highlighted issues associated with the use of correlated and non-informative variables in generating spatial and temporal projections under climate change^59,61^. Mendoza-González *et al*.^62^ provided guidelines for the selection of appropriate variables in a study similar to ours and we followed their approach. We first eliminated those variables with the highest and most significant correlation coefficients (r > 0.5 and *P* < 0.001) determined using Spearman correlations. We then used Principal Component Analysis (PCA) to assess the relative importance of the non-correlated variables that explained the highest variance within the current climatic niche of each species based on the occurrence data. As a result different variables were selected for modelling each species distribution (Supplementary Information Table S1).

We projected current distributions to 2050 (average for 2041–2060) and 2070 (average for 2061–2080) scenarios according to the Fifth Assessment Report (AR5) of the Intergovernmental Panel on Climate Change (IPCC). The AR5 assessment uses Representative Concentration Pathways (RCPs) to address the uncertainty in climate projections due to future rates of greenhouse gas and aerosol emissions and levels of stratospheric ozone. RCPs refer to different levels of radiative forcing projected for the year 2100 (2.6, 4.5, 6, and 8.5 W/m^2^). The radiative forcing is defined as the imbalance in long wave and solar radiation caused by changes in greenhouse gases and aerosols relative to preindustrial conditions.

A review on climate projections in ecological studies recommends that choosing a high and low emissions scenario is the best way of capturing the range uncertainty of emissions^63^. The climate model responses to the various RCPs are similar until mid-century, and then they begin to diverge. The use of RCP 4.5 and 8.5 effectively captures this divergence in response and the uncertainty involved, therefore we selected these RCPs representing the most plausible low-end (RCP 4.5) and the extreme high-end (RCP 8.5) estimates. The increases in global mean temperatures projected for 2100 (relative to 1990) are 1.0–2.6 °C for RCP4.5, and 2.6–4.8 °C for RCP8.5.

We used Global Climate Models (GCMs, also referred to as General Circulation Models) from four different laboratories: Met Office Hadley Centre (HadGEM2-AO), Japan Agency for Marine-Earth Science and Technology (MIROC-ESM), NASA Goddard Institute for Space Studies (GISS-E2-R), and Centre National de Recherches Meteorologiques (CNRM-CM5). We selected the GCMs with the least deviation from the mean of all the models considered in a regional assessment for Mexico^64^.

We downloaded the bioclimatic data for 16 different scenarios (4 GCMs X 2 RCPs X 2 time periods) from the WorldClim project^60^ that contains downscaled IPCC-AR5 data at the same resolution as the current climate data (1 km^2^). This resolution captures variability in topographic features in our study area, highlighting the difference between large valley bottoms and ridge tops, and allows a better prediction under climate change scenarios^65^.

### Ecological niche modelling

We used two ecological niche-modelling algorithms, Maxent^66^ and GARP^67^, to characterize species’ climatic niches for current conditions and project them to layers of selected potential future scenarios.

Maxent, maximum entropy modelling, estimates the ecological niche of species based on the location of maximum entropy distributions. We used the default Maxent program settings (version 3.3.3), except for the “extrapolation” and “clamping” options, which were disabled to avoid unrealistic extrapolations in the extreme values of the bioclimatic variables.

GARP, the genetic algorithm for rule-set production, searches iteratively for non-random correlations between species presence and environmental parameter values using several different rules. The algorithm selects rules mimicking a DNA evolution model (*e.g*., deletion, mutation) for building species prediction models. We ran 100 models and selected the ten best models following the best subsets procedure^68^.

We compared the performance of the models produced with Maxent and GARP employing the partial AUC (Area Under the Curve) ratio with the computer program Partial ROC^69^. Partial ROC evaluates the models in terms of statistical significance by generating null expectations and it is an accepted measure of model evaluation^70^ and can be used to evaluate both GARP and Maxent models effectively.

We used the algorithm that performed the best for current conditions to model future distributions. We used all the occurrence data points for modelling future conditions.

Finally, we created maps summarizing the changes between current and future potential distributions using the python-based SDMtoolbox^71^ in ESRI^®^ ArcGIS 10.2. We also used ArcGIS 10.2 to create maps of overlapping areas between *L. nivalis* and all agaves, and maps of patterns of agave richness under current and future scenarios.

## Supplementary information

GomezRuiz&LacherSupplementaryInfo

## Data Availability

The datasets generated during and/or analyzed during the current study are available from the corresponding author on reasonable request.
