# Author Correction: Climate change, range shifts, and the disruption of a pollinator-plant complex

**DOI:** 10.1038/s41598-019-53670-9

**Published:** 2019-11-20

**Authors:** Emma P. Gómez-Ruiz, Thomas E. Lacher

**Affiliations:** 1 Universidad Autónoma de Nuevo León, Facultad de Ciencias Biológicas, Ave. Universidad S/N, San Nicolás de los Garza, Nuevo León, MX 66455 Mexico; 2 Department of Wildlife and Fisheries Sciences, Texas A&M University, 534 John Kimbrough Blvd., TAMU 2258, College Station, TX 77843-2258 USA

Correction to: *Scientific Reports* 10.1038/s41598-019-50059-6, published online 01 October 2019

The Acknowledgements section in this Article is incomplete.

“We thank Socorro Gonzalez, Martha Gonzalez, Marcela Gonzalez and Ismael Cabral for their support with validating agave occurrence data and taxonomic identification. We thank Sahotra Sarkar and Octavio Rojas-Soto for their advice on species distribution models, and Adrian Castellanos for his advice on model evaluation. Especies, Sociedad y Habitat, A. C. provided support for field sampling. During the completion of this study, EPG was funded by CONACYT (CVU/SICOB: 242009/215206). Research was funded by the Mohamed bin Zayed Species Conservation Fund, Bat Conservation International, the National Park Service, Cleveland Metroparks Zoo, Cleveland Zoological Society, the American Society of Mammalogists, the Global Biodiversity Information Facility (Young Researcher Award). We thank two anonymous referees for helpful comments that improved the paper.”

should read:

“We thank Socorro Gonzalez, Martha Gonzalez, Marcela Gonzalez and Ismael Cabral for their support with validating agave occurrence data and taxonomic identification. We thank Sahotra Sarkar and Octavio Rojas-Soto for their advice on species distribution models, and Adrian Castellanos for his advice on model evaluation. Especies, Sociedad y Habitat, A. C. provided support for field sampling. During the completion of this study, EPG was funded by CONACYT (CVU/SICOB: 242009/215206). Research was funded by the Mohamed bin Zayed Species Conservation Fund, Bat Conservation International, the National Park Service, Cleveland Metroparks Zoo, Cleveland Zoological Society, the American Society of Mammalogists, the Global Biodiversity Information Facility (Young Researcher Award). We thank two anonymous referees for helpful comments that improved the paper. The open access publishing fees for this article have been partially covered by the Universidad Autónoma de Nuevo León and the Texas A&M University Open Access to Knowledge Fund (OAKFund), supported by the University Libraries and the Office of the Vice President for Research.”

