# Supplementary material for: Climate change, range shifts, and the disruption of a pollinator-plant complex
Source: Sci Rep. 2019 Oct 1;9:14048. doi: 10.1038/s41598-019-50059-6 (PMC6773846; doi:10.1038/s41598-019-50059-6)

**Title**: Climate change, range shifts, and the disruption of a pollinator-plant complex

Emma P. Gómez-Ruiz*^1^ and Thomas E. Lacher^2^

1. Universidad Autónoma de Nuevo León, Ave. Universidad S/N, San Nicolás de los Garza, Nuevo León, MX 66455, [emma.gomezrz@uanl.edu.mx](mailto:emma.gomezrz@uanl.edu.mx), Ph. +52 (81) 8329 4110
2. Department of Wildlife and Fisheries Sciences, Texas A&M University, 534 John Kimbrough Blvd., TAMU 2258, College Station TX 77843-2258, USA., [tlacher@tamu.edu](mailto:tlacher@tamu.edu), Ph. (979) 845 5750

*Corresponding author

**Supplementary information**

Table S1. Variables for each species modelling exercise. Variables selected after the PCA analysis are shown in bold. The variables marked with an asterisk are multiplied by 100.

Figure S1. Geographic distribution of gain and loss of suitable environments for all the species under future scenarios of climate change.

| Table S1. Variables for each species modeling exercise. Variables selected after the PCA analysis are shown in bold. The variables marked with an asterisk are multiplied by 100. | | | | | |
| --- | --- | --- | --- | --- | --- |
| **Variables /*Species*** | *Agave americana* | *Agave asperrima* | *Agave gentryi* | *Agave havardiana* | *Agave horrida* |
| Annual mean temperature | **14.1-24.5** | **10.4-23** | **10.5-18.9** | **13.4-21.4** | **12-14.7** |
| Mean diurnal range | 11.1-17.8 | **12.5-19.3** | 13.6-17.3 | 12.9-16.3 | **13.9-15.62** |
| Isothermality* | **46-68** | 43-78 | 50-67 | **46-53** | **69-71** |
| Temperature seasonality* | **2069-5477** | **1028-6469** | 1653-5023 | **4654-6964** | **1621-1799** |
| Maximum temperature of the warmest month | **24.2-36.8** | **20.8-38.3** | 20.9-31.1 | **26-37.4** | **21.7-25.7** |
| Minimum temperature of the coldest month | **3-10.9** | 0-9.2 | 0-5.9 | **0-4.7** | **1.3-3.3** |
| Temperature annual range | **20.8-29.7** | 20.7-32.1 | **20.7-28.2** | 26-35.3 | **19.8-22.3** |
| Mean temperature of wettest quarter | 15.3-28.7 | **12.1-28** | **12.4-22.7** | 17.8-29.6 | **12.9-16.4** |
| Mean temperature of driest quarter | 11.2-22.5 | **9.4-18.8** | 7.6-15.5 | **10.5-13.9** | 9.6-12.1 |
| Mean temperature of warmest quarter | 16.6-29.2 | 12.7-30.7 | **13-23.5** | 18.7-29.6 | 13.9-16.6 |
| Mean temperature of coldest quarter | 11-18.6 | **7.4-17.2** | 7.2-14.6 | **6.8-12.3** | 9.6-12.1 |
| Annual precipitation | **323-976** | 199-1299 | **371-1037** | **258-484** | **406-1439** |
| Precipitation of wettest month | 57-245 | 39-317 | 66-227 | **41-93** | 86-321 |
| Precipitation of driest month | 4-16 | 3-24 | **4-22** | **3-10** | **5-12** |
| Precipitation seasonality (coefficient of variation) | **53-85** | **51-110** | **53-86** | **61-103** | **70-103** |
| Precipitation of wettest quarter | 155-503 | 93-752 | 166-553 | 117-266 | 195-906 |
| Precipitation of driest quarter | 22-69 | 15-80 | **18-78** | **12-39** | **17-38** |
| Precipitation of warmest quarter | **123-466** | 74-476 | **144-288** | **117-223** | **162-391** |
| Precipitation of coldest quarter | 22-84 | **23-86** | **30-82** | **15-50** | **20-45** |

| Table S1 (Continued). | | | | | |
| --- | --- | --- | --- | --- | --- |
| **Variables /*Species*** | *Agave inaequidens* | *Agave palmeri* | *Agave parryi* | *Agave salmiana* | *Leptonycteris nivalis* |
| Annual mean temperature | **12-26** | 96-19.5 | 77-21.4 | **11.1-21.2** | 7.8-22.8 |
| Mean diurnal range | **12-17.3** | **12.8-20.7** | **15.3-20.6** | **13-17.8** | **12.7-19** |
| Isothermality* | 58-74 | 43-53 | 43-62 | 57-72 | 46-76 |
| Temperature seasonality* | **919-2959** | 5635-7486 | **3474-7770** | 1527-5018 | **1191-7381** |
| Maximum temperature of the warmest month | 22-34.6 | **27.7-37.7** | **25.3-41** | **20.8-34.9** | **17.9-34.9** |
| Minimum temperature of the coldest month | 0.6-17.7 | **-9.1-3.1** | **-11-2.8** | 0.7-9.1 | **-4.2-11.6** |
| Temperature annual range | 17-25.4 | 29.2-38.8 | 27.4-39.5 | 19.4-31 | 19.2-38.4 |
| Mean temperature of wettest quarter | 13-25.4 | 18.1-26.5 | **15.5-31.1** | 11.6-24.1 | 9.2-26.5 |
| Mean temperature of driest quarter | 9.5-26.1 | 12.6-21.5 | **7.7-24.9** | 9.1-18.6 | **6.7-22.8** |
| Mean temperature of warmest quarter | 13-27.2 | **18.6-27.3** | 16.1-31.5 | 13.1-25 | 9.9-27.4 |
| Mean temperature of coldest quarter | 9.1-24.8 | 1.2-11.8 | -1.1-11.8 | 8.9-18.6 | 4.9-20.2 |
| Annual precipitation | **816-1489** | 249-672 | 162-752 | 359-1264 | **240-1475** |
| Precipitation of wettest month | 157-372 | **58-148** | **28-192** | **65-256** | 45-360 |
| Precipitation of driest month | **2-11** | 3-13 | 2-12 | **1-18** | **2-28** |
| Precipitation seasonality (coefficient of variation) | 83-108 | **57-104** | 34-111 | **59-112** | **45-113** |
| Precipitation of wettest quarter | 440-944 | 145-327 | **63-480** | 166-708 | **106-870** |
| Precipitation of driest quarter | 17-60 | **12-48** | 10-53 | 12-58 | 11-99 |
| Precipitation of warmest quarter | **188-689** | **120-291** | 50-435 | 128-338 | **85-581** |
| Precipitation of coldest quarter | **25-201** | **44-180** | **22-173** | 12-73 | **14-153** |

| 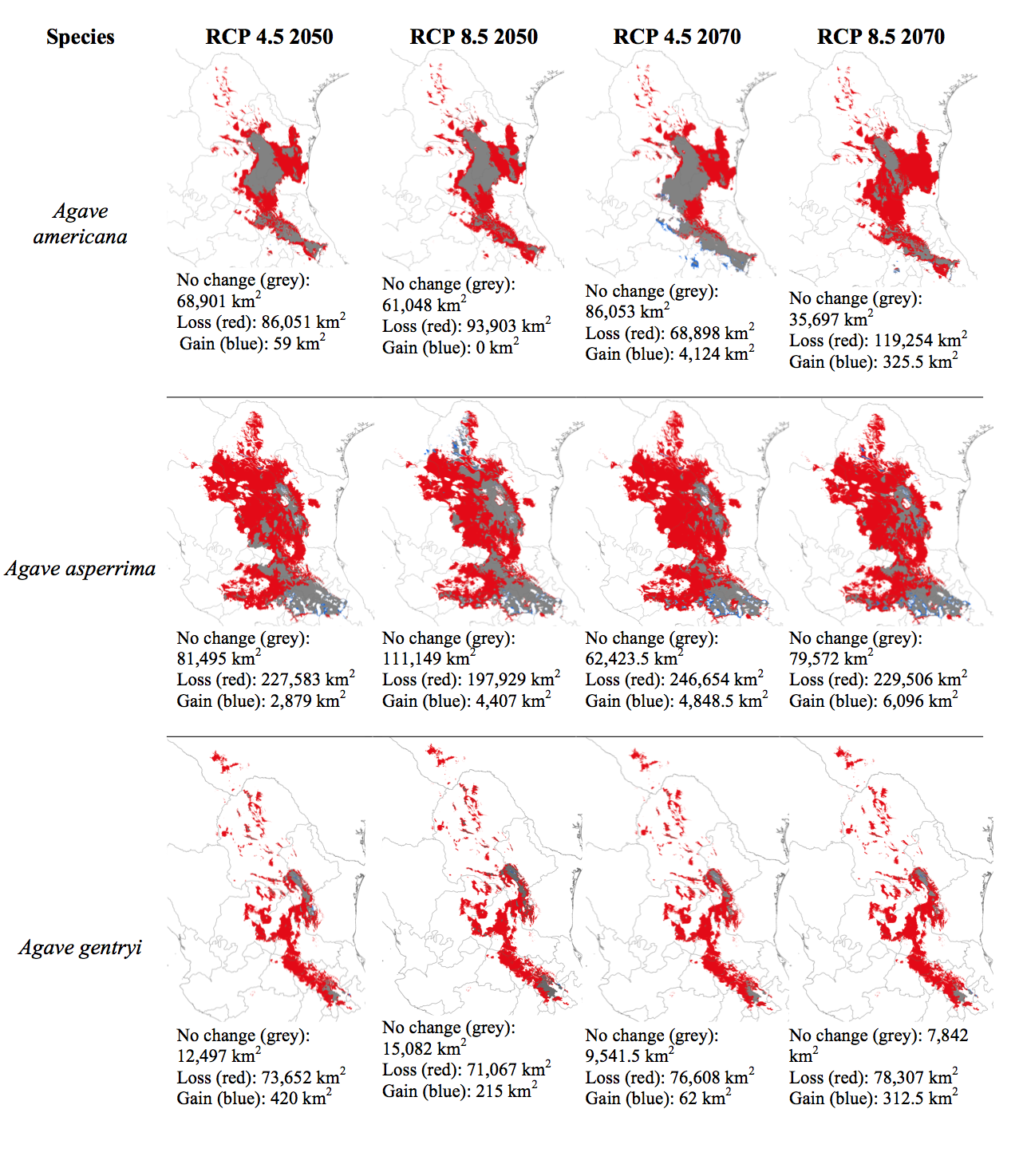 |
| --- |
| Fig. S1. Geographic distribution of gain and loss of suitable environments for all of the species under future scenarios of climate change. |

| 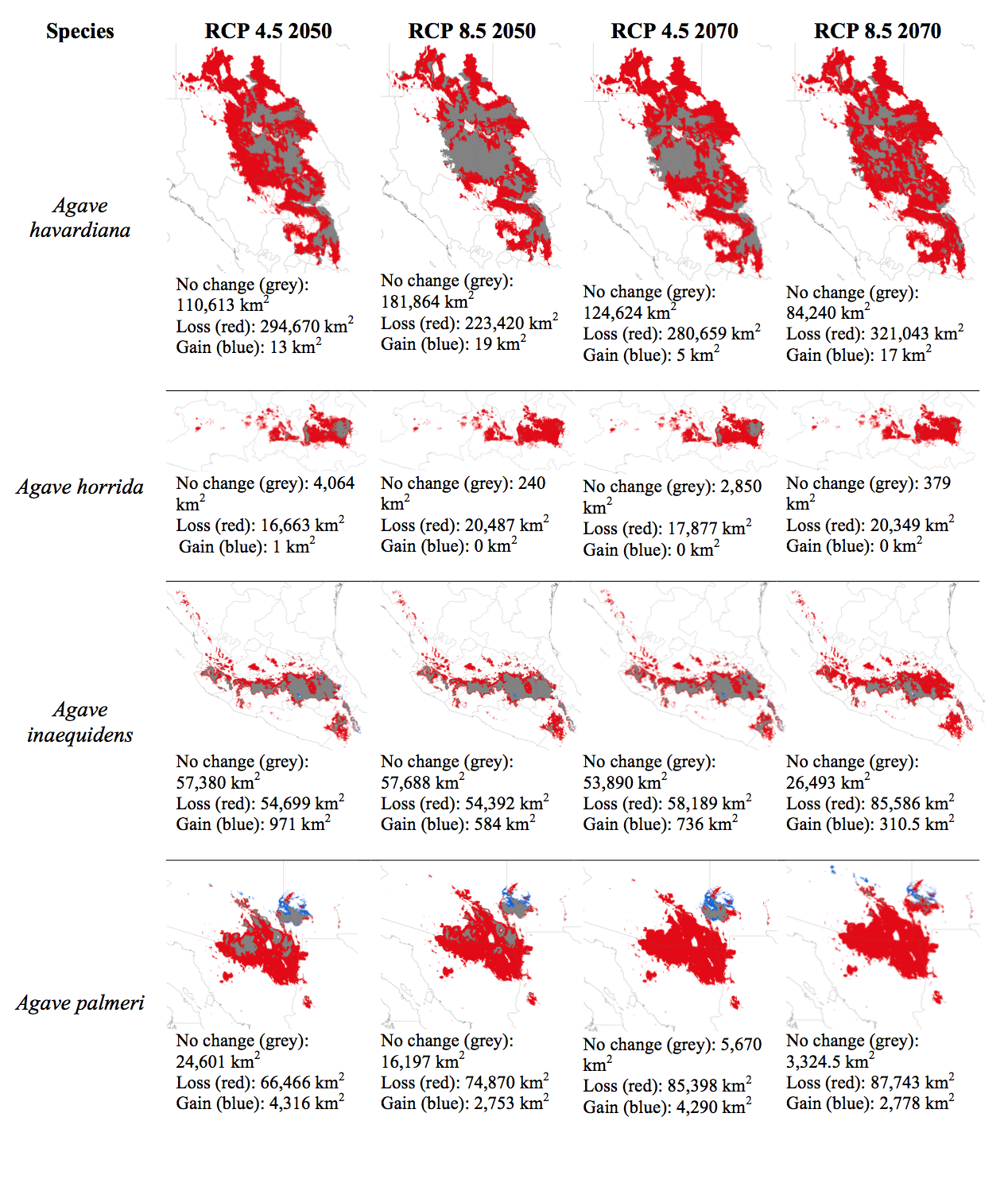 |
| --- |
| Fig. S1 (Continued). |

Fig. S1 (Continued).
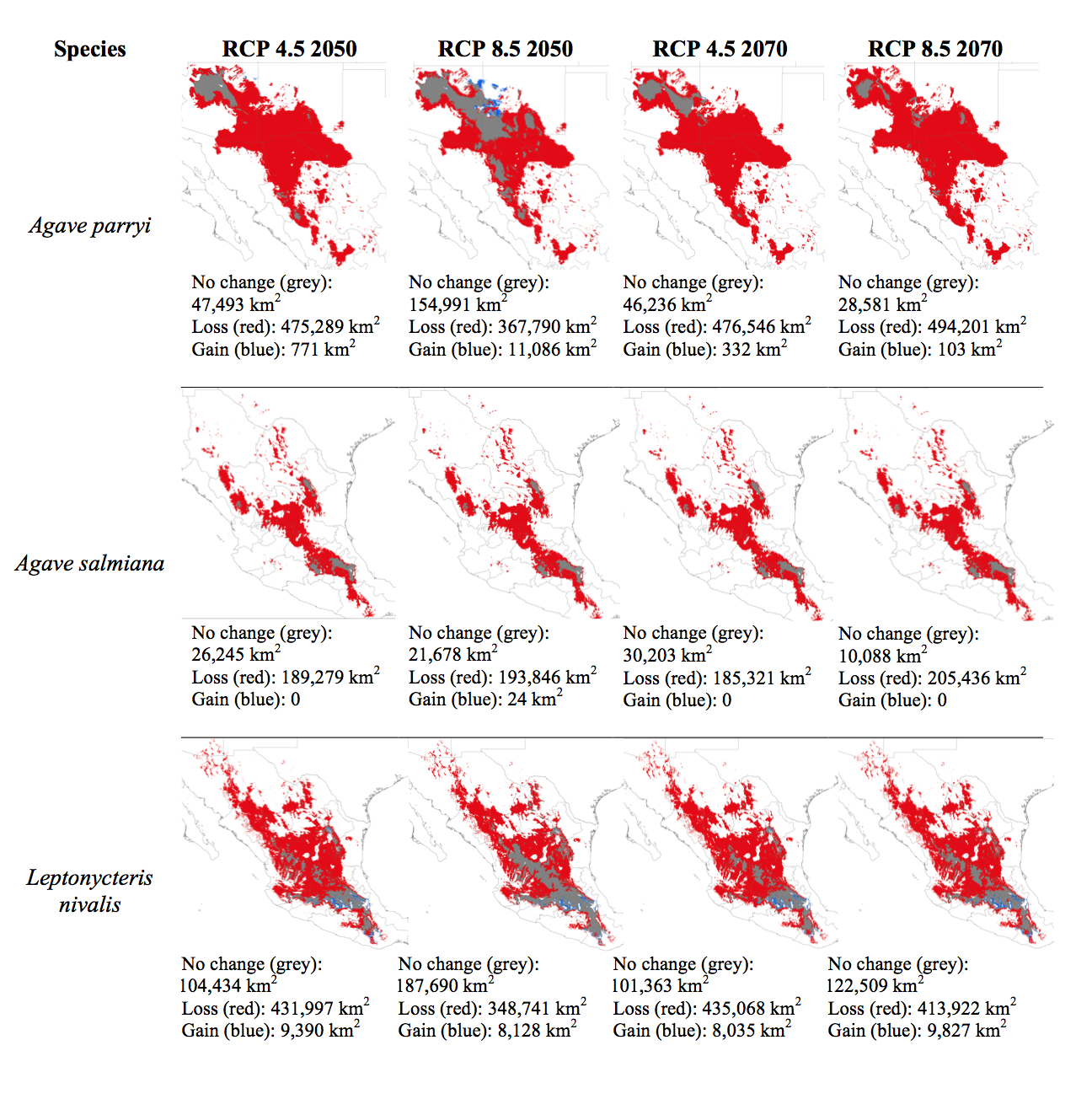

Supplement: Supplementary file 1 — GomezRuiz&LacherSupplementaryInfo [file 41598_2019_50059_MOESM1_ESM.docx]
